# Impact of Sodium, Potassium, and Calcium on the Child-Pugh and MELD Scores in Assessing the Severity of Liver Cirrhosis: A Two-Year Cross-Sectional Study

**DOI:** 10.7759/cureus.76767

**Published:** 2025-01-01

**Authors:** Twinkle Pawar, Sunil Kumar, Sourya Acharya, Rajesh Sarode, Harshitha Reddy, Avinash Parepalli, Meraj Khan, Javed Alam

**Affiliations:** 1 Internal Medicine, Jawaharlal Nehru Medical College, Datta Meghe Institute of Higher Education and Research, Wardha, IND; 2 Translational Medicine, The Hospital for Sick Children, University of Toronto, Toronto, CAN; 3 Medical AI, DigiBiomics, Mississauga, CAN

**Keywords:** calcium, child-pugh score, liver cirrhosis, meld score, potassium, sodium

## Abstract

Background and aim

Patients with chronic liver disease are prone to experiencing electrolyte imbalances as a result of physiological changes caused by cirrhosis. These imbalances have a detrimental effect on prognosis, morbidity, and mortality. This study aimed to assess the serum concentrations of sodium, calcium, and potassium in patients with liver cirrhosis and determine their correlation with disease severity and prognosis.

Methods

A cross-sectional study was conducted on 110 patients with liver cirrhosis at the Department of Medicine, Jawaharlal Nehru Medical College (JNMC), Datta Meghe Institute of Higher Education and Research (DMIHER) (DU), Wardha, Maharashtra, India, between December 2020 and November 2022. All patients diagnosed with liver cirrhosis, aged 18 years or older, were categorized into three groups: Child-Pugh class A (n = 5), class B (n = 39), and class C (n = 66).

Results

Our investigation found a notable significant disparity in serum sodium levels across groups A, B, and C, with the Child-Pugh class A group exhibiting the highest median serum sodium levels. The serum sodium < 137 mg/dL group had the highest median model for end-stage liver disease (MELD) score, and there was a statistically significant difference in the MELD score among the three groups. The distribution of serum potassium levels and results exhibited substantial variation across the groups.

Conclusion

The integration of sodium, potassium, and calcium levels into predictive models is imperative for accurately forecasting in-hospital mortality among patients with cirrhosis. These electrolytes play vital roles in physiological processes, and their inclusion enhances the predictive power of models, providing clinicians with more precise risk assessments. By incorporating these key variables, healthcare professionals can better tailor interventions and optimize patient care strategies, ultimately improving the outcomes for individuals with cirrhosis.

## Introduction

Cirrhosis occurs after prolonged injury to the liver, resulting in an alteration of its normal lobular architecture. This was marked by the production of fibrosis and nodules. The liver can sustain damage from various factors, including viruses, toxins, drugs, alcohol, genetic abnormalities, and autoimmune mechanisms. Following each injury, the liver undergoes fibrosis, but it initially maintains its function. The majority of the liver tissue undergoes fibrosis as a result of protracted injury, leading to impaired function and the onset of cirrhosis. The prevalence of liver cirrhosis is unknown worldwide; however, it is estimated to vary from 0.15% to 0.27% in the United States [1]. In 2016, liver cirrhosis and other chronic liver diseases (CLDs) accounted for 2.1% of all deaths in India [2]. In India, CLDs are typically diagnosed late in the clinical course and most frequently after decompensation has already begun, making early identification and management challenging [3].

In developing nations, the primary causes of cirrhosis are hepatitis C virus (HCV), alcoholic liver disease, and nonalcoholic steatohepatitis (NASH). In developed countries, the main causes are hepatitis B virus (HBV) and HCV infections [4]. Additional causes of cirrhosis include drug-induced liver cirrhosis, primary biliary cholangitis, alpha-1 antitrypsin deficiency, Budd-Chiari syndrome, primary sclerosing cholangitis, hemochromatosis, Wilson disease, and chronic right-sided heart failure. Cryptogenic cirrhosis is a term used to describe cirrhosis of an unknown origin. Hyponatremia in cirrhosis is defined as a serum sodium level of less than 130 mEq/L [5]. Based on specific calculations, individuals with cirrhosis and ascites had a higher probability of having <135, 130, and 120 mEq/L serum sodium levels. The likelihood of having levels below these thresholds was estimated to be 49.4%, 21.6%, and 1.2%, respectively [6]. In cirrhotic individuals, both loss of extracellular fluid and the presence of excessive fluid volume are risk factors for hyponatremia. An elevated volume of extracellular fluid characterizes hyponatremia due to the inability of the kidneys to eliminate water without solutes in proportion to the amount of pure water absorbed. Moreover, people with CLD are susceptible to fluid and electrolyte imbalances. The most frequently observed electrolyte imbalances are hyperkalemia, hyponatremia, respiratory alkalosis, and metabolic acidosis. These imbalances often accompany fluid retention, leading to edema and ascites [7].

Patients with advanced liver cirrhosis often encounter hyperkalemia, a disorder marked by disrupted potassium balance. The prevalence of this condition is higher among individuals with cirrhosis, affecting approximately 12%-14% of them, as opposed to a range of 2.1%-7.0% in the general population [8]. Reduced renal excretion is the primary cause of hyperkalemia in cirrhotic patients. This also explains why it often co-occurs with increased creatinine and blood urea nitrogen levels in patients with azotemia [9]. Hyperkalemia can also result from rhabdomyolysis caused by alcohol, gastrointestinal bleeding, or hemolysis in patients with cirrhosis. Calcium signaling imbalance is critical in liver ailments such as nonalcoholic fatty liver disease (NAFLD) and cholestasis. Calcium affects metabolism, gene expression, and cell survival, which helps the liver regenerate and operate. In NAFLD, excessive triglyceride buildup promotes inflammation and stress, which leads to fibrosis and liver damage. In cholestasis, bile flow irregularities alter calcium signaling, resulting in hepatotoxicity and inflammation. Calcium abnormalities in NAFLD and cholestasis contribute to liver disease development and metabolic dysregulation, ultimately leading to cirrhosis. Prior research has indicated that low sodium levels are an autonomous prognosticator of mortality and are associated with the progression of liver disease [10]. Potassium imbalance is a common electrolyte issue, and a U-shaped relationship exists between elevated potassium levels and unfavorable outcomes. Unfavorable consequences are commonly linked to both low potassium levels (hypokalemia) and high potassium levels (hyperkalemia) [11]. Although it is possible to assess electrolyte function in liver illnesses using relevant and insightful data, the connection between electrolyte changes and the progression of hepatic fibrosis is not readily evident. Moreover, data regarding alterations in electrolyte levels within cirrhotic liver are scarce. Thus, the present study highlights the evaluation of electrolytes like sodium, potassium, and calcium levels in patients with liver cirrhosis along with its outcome as a prognostic marker using the Child-Pugh-Turcotte (CPT) score and model for end-stage liver disease (MELD).

## Materials and methods

This study adopted a cross-sectional design to investigate the impact of sodium, potassium, and calcium levels on the assessment of liver cirrhosis severity using the Child-Pugh and MELD scores.

Study design and patient selection

**Figure 1:**
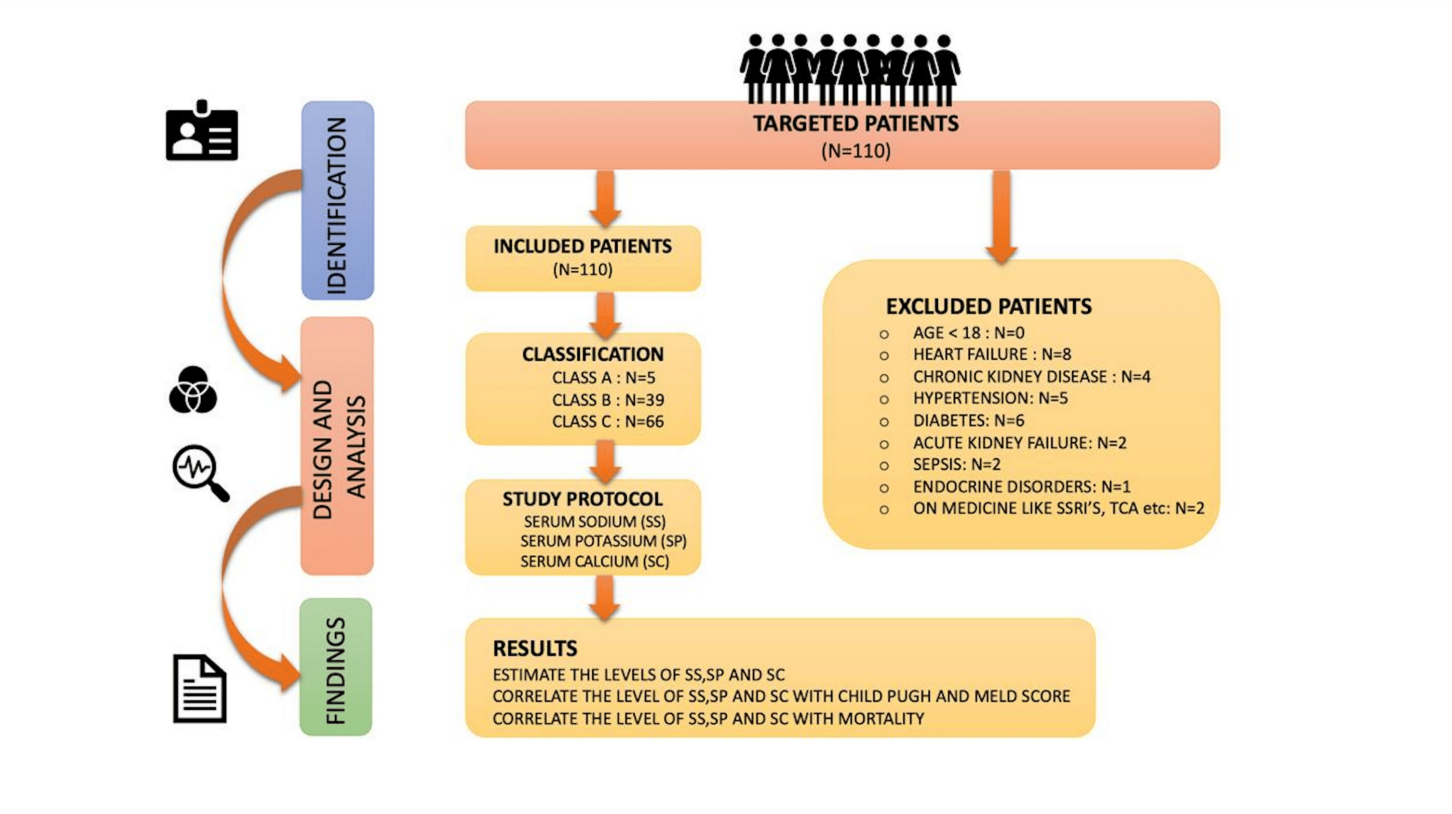
Flow chart of the study SSRI: selective serotonin reuptake inhibitor; TCA: tricyclic antidepressant Figure by Dr. Avinash Parepalli

A prospective cross-sectional study was conducted from December 2020 to November 2022 with a total of 110 patients. The study was initiated after receiving approval from the Institutional Ethical Committee with the approval letter (DMIMS (DU)/IEC/DEC 2020-21/9286). The study included all patients aged >18 years who were diagnosed with liver cirrhosis using ultrasonography in the medicine department of a rural teaching hospital in central India. The screening of patients and the study design are shown in Figure 1.

The study excluded patients with heart failure, chronic kidney disease, hypertension, diabetes, acute kidney failure, sepsis, and endocrine disorders and those taking medications such as selective serotonin reuptake inhibitors (SSRIs), tricyclic antidepressants (TCAs), monoamine oxidase (MAO) inhibitors, and cytotoxic drugs. All patients with a medical history and physical examination findings consistent with liver cirrhosis were included in this study. Upon admission, a comprehensive set of standard investigations was conducted, including a complete blood count, kidney function tests, liver function tests, and prothrombin time with an international normalized ratio (INR). Abdominal ultrasonography was then performed. Liver cirrhosis was diagnosed based on clinical examination, test results, and ultrasound findings. Patients were enrolled in the trial after applying the exclusion criteria. The CPT score at admission was determined by considering total bilirubin, albumin, INR, prothrombin time, ascites, and hepatic encephalopathy (HE) [12]. The MELD score was also calculated with serum creatinine, serum bilirubin, and INR [13]. The patients’ results were evaluated based on their discharge status and mortality and related to sodium, potassium, and calcium levels.

Sample collection and biochemical assay

Samples were collected using aseptic techniques. Hemoglobin levels (g/dL), total leukocyte count (TLC) (/mL), and platelet count (/mL) were determined using laboratory assays. The specimen was collected in an ethylenediamine tetraacetic acid (EDTA) vial and analyzed within one hour while kept at ambient temperature. The Horiba Pentra XLR instrument (Horiba ABX SAS, Montpellier, France) was used for this purpose. The VITROS 5600 machine (Ortho Clinical Diagnostics, Raritan, NJ, USA) was used to assess and estimate several parameters, including total bilirubin (mg/dL), serum albumin (g/dL), serum glutamic pyruvic transaminase (U/L), serum glutamic oxaloacetic transaminase (U/L), serum creatinine (mg/dL), serum sodium (mmol/L), serum potassium, and serum calcium, using turbidimetry. Blood samples were obtained from the patients using strict aseptic techniques and collected in a sodium citrate container. Prothrombin time (measured in seconds) and INR were evaluated using an acute care diagnostics machine.

Statistical analysis

Data analysis was conducted using the IBM Corp. SPSS v23 software (Armonk, New York, United States). Descriptive statistics were calculated for continuous data using means and standard deviations as well as medians and interquartile ranges. Frequencies and percentages were used as the categorical variables. We determined that the data exhibited a nonnormal distribution by conducting statistical tests, such as the Shapiro-Wilk test, and visually inspecting histograms and Q-Q plots. Moreover, the mean comparison of CLD questionnaire scores in each group of Child-Pugh scores was performed using the Kruskal-Wallis test. Fisher's exact test was used because of the presence of expected frequencies below five in more than 25% of the cells in the contingency tables. Statistical significance was set at p < 0.05.

## Results

**Figure 2:**
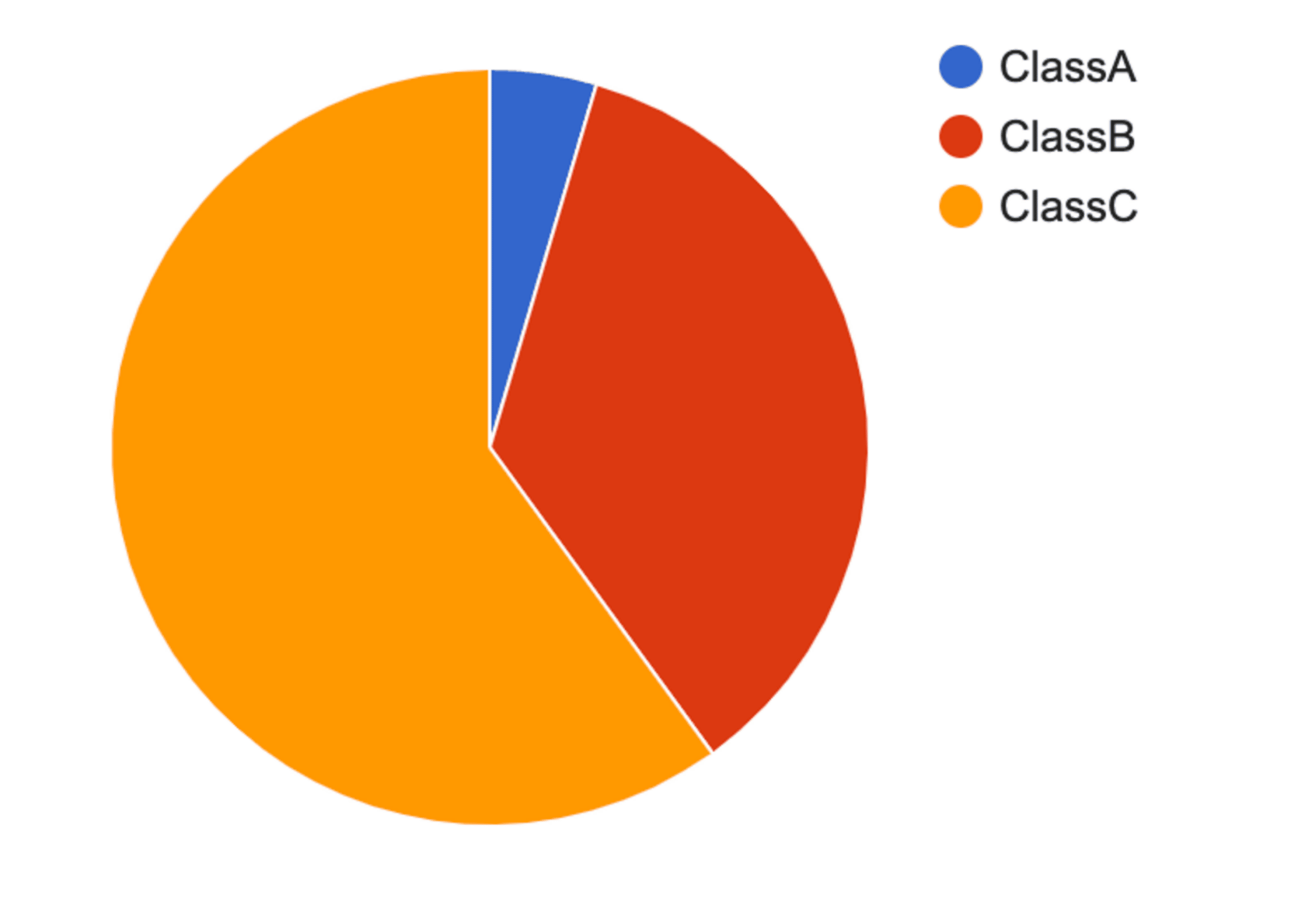
Pie chart illustrating the distribution of patients in the Child-Pugh classification Figure by Dr. Parepalli Avinash Class A: 4.55% (N = 5) Class B: 35.45% (N = 39) Class C: 60% (N = 66)

Our study included 110 individuals diagnosed with liver cirrhosis, of whom 56 were aged between 41 and 60 years. Among this age group, 93 were male patients and 17 were female patients, as shown in Figure 2.

Among the 110 patients diagnosed with cirrhosis of the liver, 92 had a documented history of alcoholism, and 12 had viral hepatitis. Among the 110 patients, 71 exhibited hyponatremia, 83 had normal potassium levels, and 90 had hypocalcemia. The average TLC was 10,157, the average bilirubin level was 4.6, the average serum albumin level was 2.5, and the average INR was 1.76. Additional baseline characteristics and investigations are presented in Tables 1, 2. 

**Table 1:** Baseline characteristics among study groups H/O: history of; SD: standard deviation; BPM: beats per minute; mmHg: millimeters of mercury; BP: blood pressure

Parameters	Mean ± SD || frequency (%)
Age	
18-40 years	43 (39.1%)
41-60 years	56 (50.9%)
>60 years	11 (10.0%)
Gender	
Male	93 (84.5%)
Female	17 (15.5%)
H/O alcoholism (yes)	92 (83.6%)
H/O viral hepatitis (yes)	12 (10.9%)
Cryptogenic cirrhosis (yes)	6 (5.5%)
Pulse (BPM)	87.08 ± 12.40
Systolic BP (mmHg)	112.04 ± 15.18
Diastolic BP (mmHg)	70.84 ± 8.04

**Table 2:** Investigation of study patients TLC: total leukocyte count; SGPT: serum glutamic pyruvic transaminase; SGOT: serum glutamic oxaloacetic transaminase; INR: international normalized ratio; SD: standard deviation; g/dL: grams/deciliter; mm³: cubic millimeters; mg/dL: milligrams/deciliter; s: seconds: U/L: units/liter; mmol/L: millimoles/liter

Investigations	Reference value	Mean ± SD || frequency (%)
Hemoglobin (g/dL)	3.5-17.5 g/dL (males); 12.0-15.5 g/dL (females)	8.67 ± 2.18
TLC (/mm³)	4,000–10,000/mm³	10,157.27 ± 8,597.15
Platelet count (/mm³)	150,000–450,000/mm³	134,316.36 ± 112,979.63
Serum creatinine (mg/dL)	0.6-1.4 mg/dL	1.48 ± 1.03
Serum sodium	136-144 mmol/L	
<137		71 (64.5%)
137-145		34 (30.9%)
>145		5 (4.5%)
Serum potassium	3.7-5.1 mmol/L	
<3.5		13 (11.8%)
3.5-5.1		83 (75.5%)
>5.1		14 (12.7%)
Serum calcium	8.5-10.2 mg/dL	
<8.4		90 (81.8%)
8.4-10.2		19 (17.3%)
>10.2		1 (0.9%)
Total bilirubin (mg/dL)	0.1-1.2	4.66 ± 4.45
SGPT (U/L)	0-40	66.70 ± 193.46
SGOT (U/L)	0-40	99.18 ± 98.54
Serum albumin (g/dL)	3.5-5.0	2.58 ± 0.63
Prothrombin time (s)	11-13.5	21.31 ± 8.91
INR	0.8-1.1	1.76 ± 0.79

In the present study, 66 patients were classified as having Child-Pugh class C. Of these, 47 (71.2%) had hyponatremia, 47 (71.2%) had normal potassium levels, and 53 (80.3%) had hypocalcemia. However, these differences were not significant as shown in Table 3.

**Table 3:** Comparative analysis and correlation of Child-Pugh classification with serum concentrations of sodium, potassium, and calcium Fisher's exact test was used in the analysis p < 0.05 is statistically significant

Parameters	Child-Pugh class			p-value
	A (n (%) = 5)	B (n (%) = 39)	C (n (%) = 66)	
Serum sodium				0.077
<137	1 (20.0%)	23 (59.0%)	47 (71.2%)	
137-145	3 (60.0%)	14 (35.9%)	17 (25.8%)	
>145	1 (20.0%)	2 (5.1%)	2 (3.0%)	
Serum potassium				0.856
<3.5	0 (0.0%)	4 (10.3%)	9 (13.6%)	
3.5-5.1	5 (100.0%)	31 (79.5%)	47 (71.2%)	
>5.1	0 (0.0%)	4 (10.3%)	10 (15.2%)	
Serum calcium				0.948
<8.4	4 (80.0%)	33 (84.6%)	53 (80.3%)	
8.4-10.2	1 (20.0%)	6 (15.4%)	12 (18.2%)	
>10.2	0 (0.0%)	0 (0.0%)	1 (1.5%)	

Among the 110 patients with liver cirrhosis, there was a notable difference among the three groups in relation to the MELD score (p < 0.001). The hyponatremia group had the highest median MELD score, and there were no significant differences between the groups in terms of serum potassium and MELD scores (p = 0.388). Similarly, there were no significant differences between the groups in terms of serum calcium level and MELD score (p = 0.297), although patients with low calcium (<8.4) had a low median MELD score (20.22 ± 8.85), as shown in Table 4.

**Table 4:** Correlation and association between MELD scores and serum concentrations of sodium, potassium, and calcium Fisher's exact test was used for the analysis SD: standard deviation; MELD: model for end-stage liver disease p < 0.05 is considered statistically significant

Parameters	MELD score (mean ± SD)	p-value
Serum sodium		<0.001
<137	23.23 ± 8.86	
137-145	15.03 ± 7.20	
>145	14.40 ± 7.02	
Serum potassium		0.388
<3.5	21.92 ± 11.27	
3.5-5.1	19.55 ± 8.78	
>5.1	23.14 ± 9.17	
Serum calcium		0.297
<8.4	20.22 ± 8.85	
8.4-10.2	19.84 ± 10.37	
>10.2	35.00 ± 0	

ROC curve analysis

**Figure 3:**
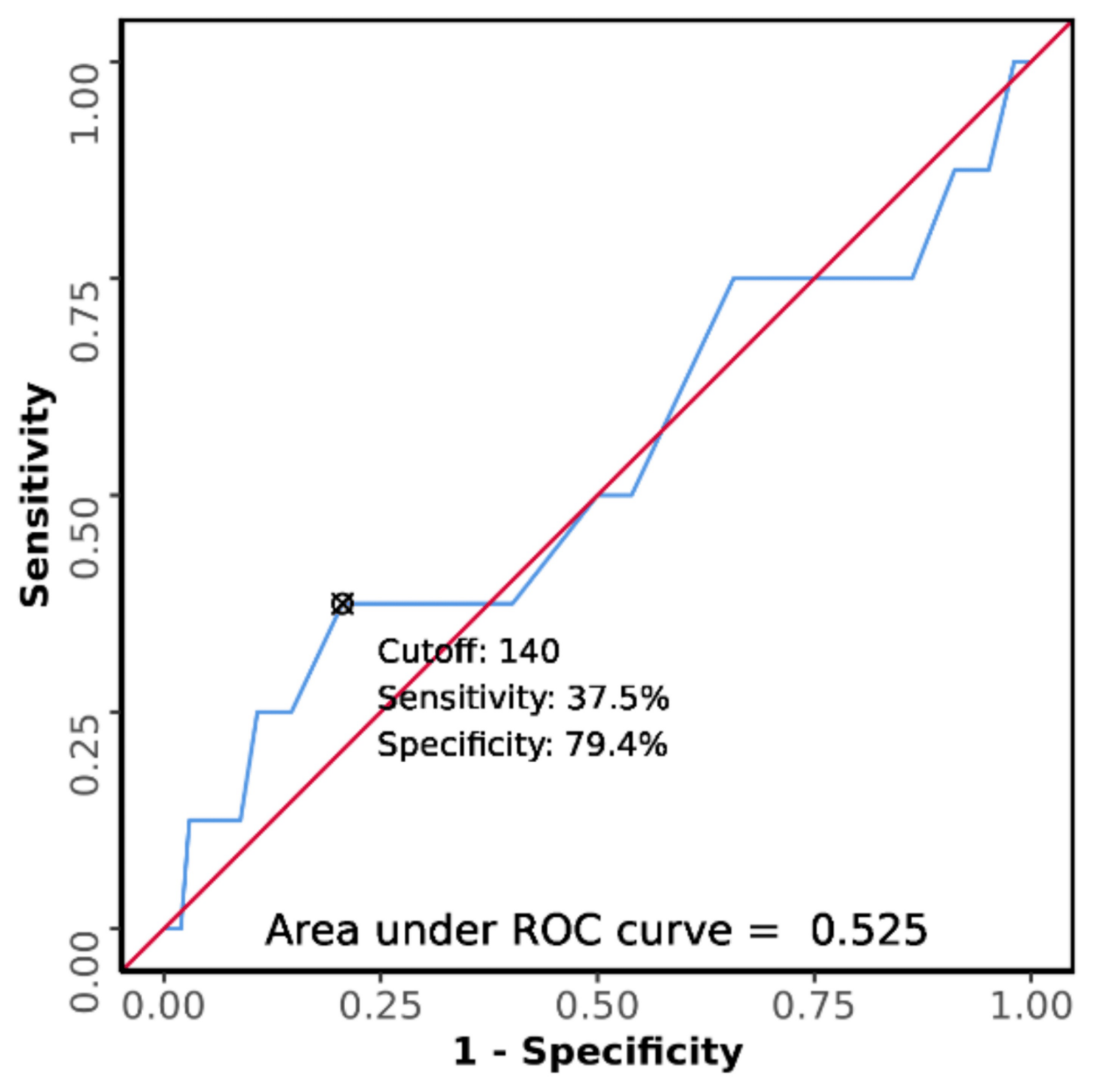
Receiver operating characteristic (ROC) curve analysis was conducted to evaluate the diagnostic performance of serum sodium

The area under the receiver operating characteristic (ROC) curve for serum sodium predicting the outcome (death vs. discharge) was 0.525 (95% CI: 0.275-0.775), thus demonstrating poor diagnostic performance. This difference was not statistically significant (p = 0.818). At a cutoff of serum sodium ≥ 140, it predicts the outcome: death with a sensitivity of 38% and specificity of 79% as shown in Figure 3.

**Figure 4:**
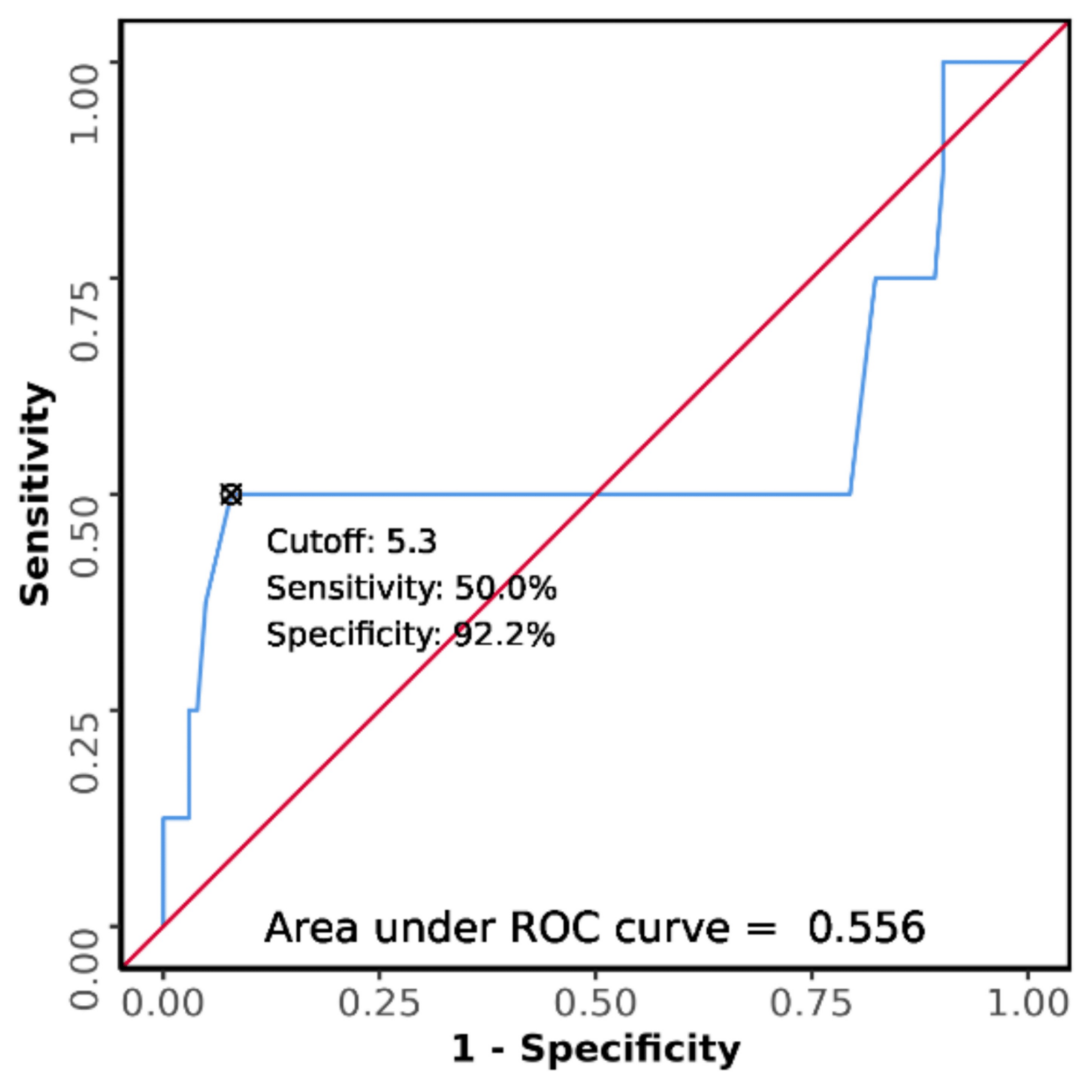
Receiver operating characteristic (ROC) curve analysis was conducted to evaluate the diagnostic performance of serum potassium

The area under the ROC curve for serum potassium predicting the outcome (death vs. discharge) was 0.556 (95% CI: 0.249-0.863), thus demonstrating poor diagnostic performance. This difference was not statistically significant (p = 0.604). At a cutoff serum potassium level ≥ 5.3, it predicts the outcome: death with a sensitivity of 50% and a specificity of 92% as shown in Figure 4.

**Figure 5:**
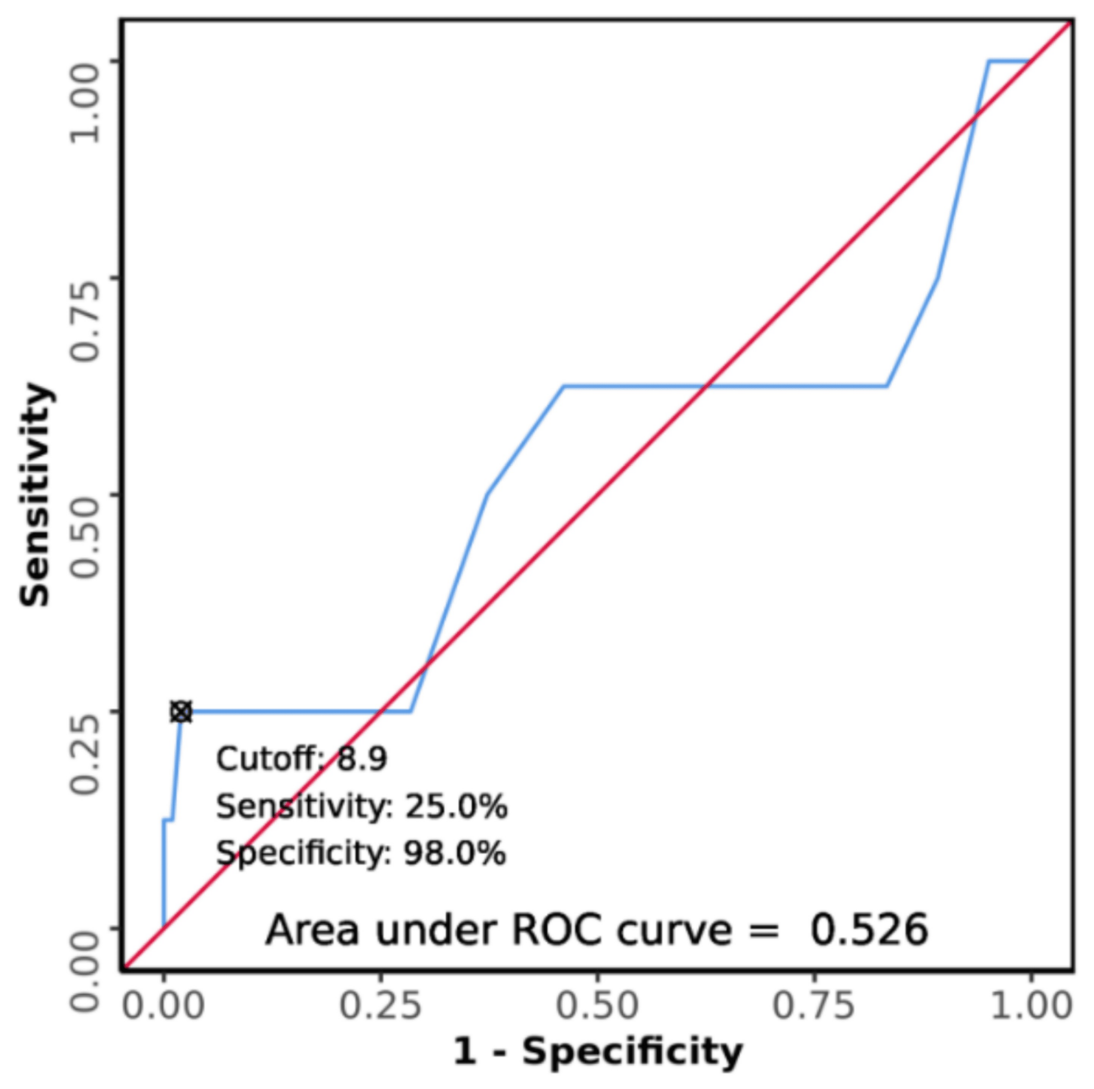
Receiver operating characteristic (ROC) curve analysis was conducted to evaluate the diagnostic performance of serum calcium

Poor diagnostic performance was shown by the area under the ROC curve for serum calcium predicting outcome: death vs. outcome: discharge, which was 0.526 (95% CI: 0.256-0.795). This difference was not statistically significant (p = 0.813). At a cutoff of serum calcium ≥ 8.9, it predicts the outcome: death with a sensitivity of 25% and a specificity of 98%, as shown in Figure 5.

## Discussion

This prospective cross-sectional study aimed to assess the significance of serum sodium, potassium, and calcium levels in predicting the severity of cirrhosis in terms of Child-Pugh and MELD scores, as well as the likelihood of in-hospital death in cirrhotic patients. We selected 110 individuals with liver cirrhosis for this study, of which 66 were classified as Child-Pugh class C. Out of these, 71.2% had a sodium level below 135 mEq/L, 71.2% had normal potassium levels ranging from 3.5 to 5.1 mEq/L, and 80.3% had calcium levels below 8.4 mEq/L; however, this difference was not statistically significant. In a study conducted by Singh et al., it was shown that the patients classified as class B had the greatest average levels of sodium and calcium. In contrast, patients classified as class A had the highest average potassium level, although this difference was not statistically significant [14]. In a separate study conducted by Gandhi et al., the prevalence of dyselectrolytemia (specifically hyponatremia and hypokalemia) was significantly higher in Child-Pugh classes B (76.1%) and C (90.2%) than in class A (20%) (p-value = 0.01) [13]. A study conducted by Mamun et al. revealed that approximately 30% of patients with cirrhosis had hyponatremia (serum sodium > 130 mEq/L). However, the study did not find any link or correlation between hyponatremia and the Child-Pugh score [15]. According to a study conducted by Malani et al., 20% of patients with Child-Pugh score A, 23.8% of patients with Child-Pugh score B, and 91.97% of patients with Child-Pugh score C experienced hyponatremia [16]. Among 110 patients with cirrhosis of the liver, there was a significant difference in the MELD scores between the three groups (p < 0.001), with the median MELD score being highest in the hyponatremia group. There was no significant difference in the MELD scores between the groups for serum calcium (p = 0.297) and serum potassium (p = 0.388), with the median MELD score being highest in hypercalcemia and hyperkalemia. In a research investigation, Gandhi et al. discovered that patients with MELD scores greater than 21 exhibited electrolyte disturbances (92.3%) (hyponatremia and hypokalemia) that were statistically significant (p = 0.04) [17]. Patients with lower serum sodium levels had mean MELD scores that were considerably higher in the study conducted by Singh et al. [18]. Of the 110 patients in our study, 102 were discharged, and eight experienced fatality. Among the individuals who were not alive, five had sodium levels below 135, four had potassium levels above 5.1, and six had calcium levels below 8.4. Nevertheless, there was a notable disparity between the different groups regarding the allocation of serum potassium in relation to the outcome. According to a study conducted by Cárdenas et al., patients who had both acute and chronic liver failure and hyponatremia had a significantly lower three-month survival rate compared to patients with acute-on-chronic liver failure but without hyponatremia (35.8% vs. 58.7%, respectively; p < 0.001) [19]. The investigation conducted by Majety et al. found no correlation between admission, peak blood calcium levels, and mortality [20].

Therefore, mortality rates were higher in hyponatremia, hyperkalemia, and hypocalcemia cases, but statistical significance was observed only for serum potassium levels. In conclusion, based on the results of the above study, it may not be plausible to assertively state that serum electrolytes other than sodium are important in predicting mortality in cirrhosis patients. Given the potentially limited predictive accuracy of individual constituents, validating the therapeutic relevance of serum sodium, potassium, and calcium concentrations is necessary [21,22]. The present investigation did not emphasize the association between comorbidities, which can complicate the predictive role of serum sodium, potassium, and calcium levels in liver cirrhosis. Since the current study focused exclusively on hospitalized patients with liver cirrhosis, it primarily highlights the significance of liver decompensation in these circumstances while neglecting its predictive value in compensated cases of liver cirrhosis.

Limitations

The main limitation was the small sample size, which may restrict the statistical power and applicability of the findings to larger groups. As a cross-sectional research, data was collected simultaneously, making it difficult to establish causal relationships or follow changes over time. Patients with substantial comorbidities (such as heart failure or chronic renal disease) were excluded, which may limit the data's application to patients with more complex medical histories. Conducting research at a single institution may add bias and limit the variety of the patient group, reducing the findings' generalizability. It is possible that the study did not effectively account for confounding variables, such as other underlying health issues and drugs that impact electrolyte levels. The timing of serum electrolyte measures about clinical events (e.g., hospitalization and decompensation) might impact the results and interpretation. The study discovered that serum sodium, potassium, and calcium levels had poor diagnostic performance in predicting outcomes, suggesting that these measurements alone may not accurately predict mortality risk. The absence of long-term follow-up data on patients after discharge hinders our knowledge of the long-term consequences of electrolyte abnormalities in cirrhosis patients. Addressing these limitations in future studies may improve the validity and usefulness of electrolyte imbalance results in liver cirrhosis.

## Conclusions

To accurately predict the likelihood of in-hospital mortality in patients with cirrhosis, it is imperative to incorporate the blood levels of sodium, less likely potassium, and calcium into predictive models. These electrolytes serve as critical indicators of physiological homeostasis and provide valuable insights into the disease severity and prognosis. However, further investigation is warranted to validate the clinical usefulness of serum sodium, potassium, and calcium levels in predicting outcomes in patients with cirrhosis, given the potential limitations in predictive accuracy associated with these factors. Future research should focus on refining predictive models and elucidating the optimal integration of electrolyte measurements to enhance risk stratification and improve patient care strategies for the management of cirrhosis.
